# 应用Ca^2+^荧光探针fluo-3和fluo-4测定H_2_O_2_诱导的A549细胞凋亡过程中的[Ca^2+^]_i_变化

**DOI:** 10.3779/j.issn.1009-3419.2014.03.03

**Published:** 2014-03-20

**Authors:** 四洋 张, 春艳 李, 建 高, 雪杉 邱, 泽实 崔

**Affiliations:** 1 110001 沈阳，中国医科大学实验技术中心 Department of Pathology, China Medical University, Shenyang 110001, China; 2 110001 沈阳，中国医科大学病理学教研室 Center of Laboratory Technology and Experimental Medicine, China Medical University, Shenyang 110001, China

**Keywords:** Fluo-3, Fluo-4, Ca^2+^, H_2_O_2_, 细胞凋亡, Fluo-3, Fluo-4, Ca^2+^, H_2_O_2_, Apoptosis

## Abstract

**背景与目的:**

肺癌是世界范围内常见的恶性肿瘤，Ca^2+^对于肿瘤细胞凋亡有重要的调控作用。实时监测肺癌细胞内Ca^2+^水平，有助于深入研究Ca^2+^介导肺癌细胞凋亡的分子机制。本研究旨在观察Ca^2+^荧光探针fluo-3和fluo-4在H_2_O_2_诱导的A549细胞凋亡过程中的应用，实时测定胞浆Ca^2+^浓度（[Ca^2+^]_i_），探讨[Ca^2+^]_i_与细胞凋亡的关系，并比较两种Ca^2+^探针在荧光强度及[Ca^2+^]_i_测定值方面的差异。

**方法:**

采用Ca^2+^荧光探针fluo-3和fluo-4负载细胞，1 h后用不同浓度的H_2_O_2_刺激细胞，激光扫描共聚焦显微镜实时测定选取细胞的[Ca^2+^]_i_变化。采用DAPI染色试剂盒观察H_2_O_2_刺激后细胞凋亡情况。

**结果:**

在相同的探针浓度、负载时间和相同的图像采集参数的条件下，选定细胞内fluo-4平均荧光强度高于fluo-3。50 mM H_2_O_2_刺激后，A549细胞胞浆内[Ca^2+^]_i_迅速升高，通过公式计算发现采用fluo-3探针负载的选定细胞中[Ca^2+^]_i_变化范围是112.2 nM-1, 069.6 nM，采用fluo-4探针负载的选定细胞中[Ca^2+^]_i_变化范围是7.6 nM-505.4 nM。同时发现经H_2_O_2_刺激后，凋亡细胞百分比明显增加（*P* < 0.01）。

**结论:**

H_2_O_2_促进A549细胞内Ca^2+^释放，诱导细胞凋亡。Ca^2+^探针fluo-4可能更适合于监测含量较低的细胞中[Ca^2+^]_i_变化。

## Abstract (en)

**Background and objective:**

Lung cancer is a common malignant tumor all over the world, and Ca^2+^ is a critical regulator for apoptosis of cancer cells. The monitoring of cytoplastic Ca^2+^ level in real-time will contribute to further investigate the molecular mechanisms of apoptosis mediated by Ca^2+^ in lung cancer cells. To evaluate the Ca^2+^ indicator fluo-3 and fluo-4 in the process of H_2_O_2_ induced the apoptosis of lung adenocarcinoma A549 cells. The cytoplastic Ca^2+^ concentration ([Ca^2+^]_i_) was determined in real-time, and the correlations between [Ca^2+^]_i_ and cell apoptosis were investigated. The differences in fluorescence intensity and measured value were compared between the two Ca^2+^ indicators.

**Methods:**

Cells were loaded with the Ca^2+^ indicator fluo-3 or fluo-4 for 1 h, and then stimulated with 50 mM H_2_O_2_. Laser scanning confocal microscope was applied to perform real-time monitoring on the variation of [Ca^2+^]_i_ in selected cells. DAPI staining was used to observe apoptosis in H_2_O_2_ treated cells.

**Results:**

Our results showed that the fluorescence intensity of fluo-4 was stronger than that of fluo-3 in the same condition of dye concentration, loading time and image acquisition parameters before or after H_2_O_2_ stimulation. The cytoplastic [Ca^2+^]_i_ was rapidly elevated in H_2_O_2_ stimulated A549 cells. The range of [Ca^2+^]_i_ in selected cells loaded with fluo-3 was 112.2 nM-1, 069.6 nM, and that in selected cells loaded with fluo-4 was 7.6 nM-505.4 nM. Moreover, the apoptotic rate was significantly increased in H_2_O_2_ treated cells, compared with untreated ones (P < 0.01).

**Conclusion:**

In summary, H_2_O_2_ promoted Ca^2+^ release in A549 cells, and induced cell apoptosis. Ca^2+^ indicator fluo-4 was probably more applicable to measure [Ca^2+^]_i_ in cells with less content of Ca^2+^.

Ca^2+^是非常重要的细胞内第二信使，参与肌细胞收缩、神经递质释放、细胞分化和细胞凋亡等生理、病理过程。细胞内生理Ca^2+^浓度为10 nM-100 nM，Ca^2+^超载时浓度为基础浓度的10倍左右，与心脏疾病、神经损伤、肿瘤发生等多种疾病的发生发展密切相关^[1]^。H_2_O_2_是常见的活性氧分子，向细胞外液加入H_2_O_2_，可以使细胞处于氧化应激状态，文献^[2]^报道Ca^2+^介导了H_2_O_2_作用下的大鼠胰腺腺泡AR42J细胞的凋亡。肺癌是世界范围内常见的恶性肿瘤，研究^[3-5]^表明，引起胞浆Ca^2+^浓度升高的刺激通过依赖线粒体途径的caspases通路或内质网应激途径，诱导肺癌细胞发生凋亡。实时监测肺癌细胞内Ca^2+^浓度和胞浆内Ca^2+^水平，有助于深入研究Ca^2+^介导肺癌细胞凋亡的分子机制，以寻找促进肺癌细胞凋亡的有效方法。

自1989年开始，应用荧光染料fluo-3对细胞内Ca^2+^成像，揭示了很多涉及Ca^2+^信号时空变化的细胞生物学功能。Fluo-3也结合流式细胞仪用于检测Ca^2+^作为第二信使参与信号转导和细胞药理学筛选等实验研究。Fluo-3用于监测活细胞内[Ca^2+^]_i_变化，其最主要的优势在于利用488 nm的氩离子激光源，可以在激光扫描共聚焦显微镜下观察，fluo-3与Ca^2+^结合后其荧光强度迅速增强。Fluo-4是fluo-3的衍生物，用F取代了fluo-3分子中的2个Cl。其激发波长较fluo-3更接近488 nm，产生更强、更稳定的荧光信号，适合于多数配置氩离子激光器的激光扫描共聚焦显微镜观察^[6]^，也可以用于流式细胞仪检测，以及利用多功能酶标仪读取荧光信号^[7]^。

本研究采用目前实验室常用的Ca^2+^荧光探针fluo-3和fluo-4负载人肺癌A549细胞，观察H_2_O_2_处理的A549细胞中[Ca^2+^]_i_变化，对fluo-3和fluo-4的荧光强度和[Ca^2+^]_i_测定值进行比较和分析，并探讨在H_2_O_2_作用下细胞凋亡情况。

## 1 材料与方法

### 1.1 主要试剂与仪器

Fluo-3 AM（Biotium公司，纯度95%）和fluo-4 AM（DOJINDO公司，纯度98%），均用DMSO溶解，0.22 μm滤膜过滤除菌，贮存液浓度为1 mM，-20 ℃避光保存。RPMI-1640培养基和胎牛血清为Hyclone公司产品，激光共聚焦显微镜专用培养皿（35 mm）购自NEST公司，Ionomycin和细胞固定液均为碧云天公司产品，DAPI细胞凋亡染色试剂盒购自凯基公司，H_2_O_2_为国产分析纯。

### 1.2 细胞培养

采用含10%胎牛血清的RPMI-1640培养基，于37 ℃、5%CO2培养箱中培养肺癌细胞A549，0.25%胰酶-EDTA消化传代，所有实验均采用对数生长期细胞。

### 1.3 探针负载细胞并观察[Ca^2+^]_i_变化

荧光探针fluo-3 AM和fluo-4 AM负载细胞时用无Ca^2+^细胞外液（135 mM NaCl、2 mM KCl、2 mM MgCl2、10 mM HEPES、4 g/L葡萄糖）稀释成工作浓度5 μM。细胞传代24 h后，弃去培养基，用PBS漂洗3次，向细胞中加入fluo-3（5 μM）或fluo-4（5 μM）工作液，避光37 ℃孵育40 min。弃去荧光探针，更换新的无Ca^2+^细胞外液，避光37 ℃继续孵育20 min，保证AM在细胞内被充分水解。在FV1000型激光共聚焦显微镜（Olympus, Japan）下观察选择贴壁良好、形态伸展、荧光强度较亮的细胞，设置扫描条件为488 nm波长激发、39%激光强度、PMT（790）、Pinhole（310 μm）、同时采集DIC图像，每5 s采集一幅，共采集40 min左右。首先扫描10幅待荧光强度曲线稳定后，向细胞中加入不同浓度的H_2_O_2_（5 mM、10 mM或50 mM）；曲线稳定后，向细胞中加入EGTA（4 mM）+ Ionomycin（5 μM）；曲线再一次稳定后，向细胞中加入CaCl2（10 mM），曲线稳定后中止实验。钙离子浓度计算公式为Kd(F-Fmin)/(Fmax-F)^[8]^，其中fluo-3探针Kd=400 nM，fluo-4探针Kd=360 nM。

### 1.4 DAPI染色检测细胞凋亡

细胞经H_2_O_2_处理30 min后，用细胞固定液4%多聚甲醛室温固定10 min，加入DAPI染色液避光孵育20 min，在倒置荧光显微镜下观察，随机选取5个200×镜下视野，计算凋亡细胞百分比。

### 1.5 统计学分析

采用SPSS13.0统计学软件，进行数据分析及处理。应用*t*检验比较fluo-3和fluo-4平均荧光强度的差异，应用*χ*^2^检验比较凋亡细胞百分比，*P* < 0.05为差异有统计学意义。

## 2 结果

### 2.1 未经H_2_O_2_刺激前细胞中fluo-3和fluo-4染色结果比较

在相同的细胞负载和图像采集条件下，未加H_2_O_2_处理时，fluo-3染色后的细胞在488 nm激发光激发下发出较弱的绿色荧光（图 1）。Fluo-4染色后的细胞在488 nm激发光激发下发出较强的绿色荧光（图 1）。

**1:**
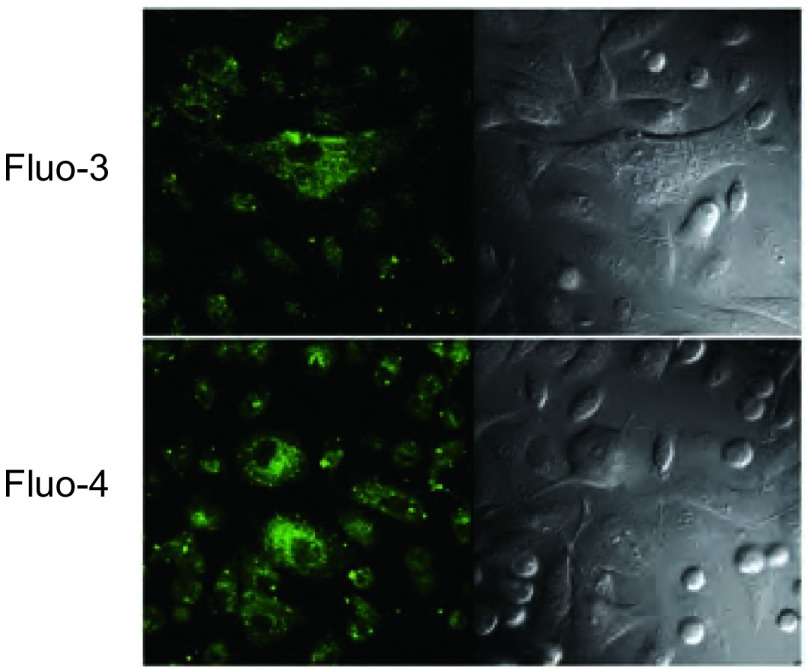
H_2_O_2_刺激前，负载fluo-3或fluo-4的细胞荧光强度比较。66 mm×58 mm（300×300 DPI）。 The comparison of fluorescence intensity in cells loaded with fluo-3 or fluo-4 before H_2_O_2_ stimulation. 66 mm×58 mm (300×300 DPI).

### 2.2 H_2_O_2_处理的细胞[Ca^2+^]_i_变化

在5 mM或10 mM H_2_O_2_作用下，fluo-3和fluo-4荧光强度均无明显变化（结果未显示）。加入50 mM H_2_O_2_后，细胞荧光强度逐渐增强（图 2），观察至40 min左右时，荧光强度曲线稳定不再变化（图 3）。在实时观察[Ca^2+^]_i_变化过程中，荧光探针fluo-3和fluo-4均未出现光漂白现象。观察结束时，fluo-3荧光强度增加至3.0倍，fluo-4荧光强度增加至2.6倍。H_2_O_2_刺激前，fluo-4荧光强度约是fluo-3荧光强度的2.0倍，而H_2_O_2_刺激后fluo-4荧光强度约是fluo-3荧光强度的1.8倍（表 1）。通过公式计算发现采用fluo-3探针负载的选定细胞中[Ca^2+^]_i_变化范围是112.2 nM-1, 069.6 nM，采用fluo-4探针负载的选定细胞中[Ca^2+^]_i_变化范围是7.6 nM-505.4 nM。

**2:**
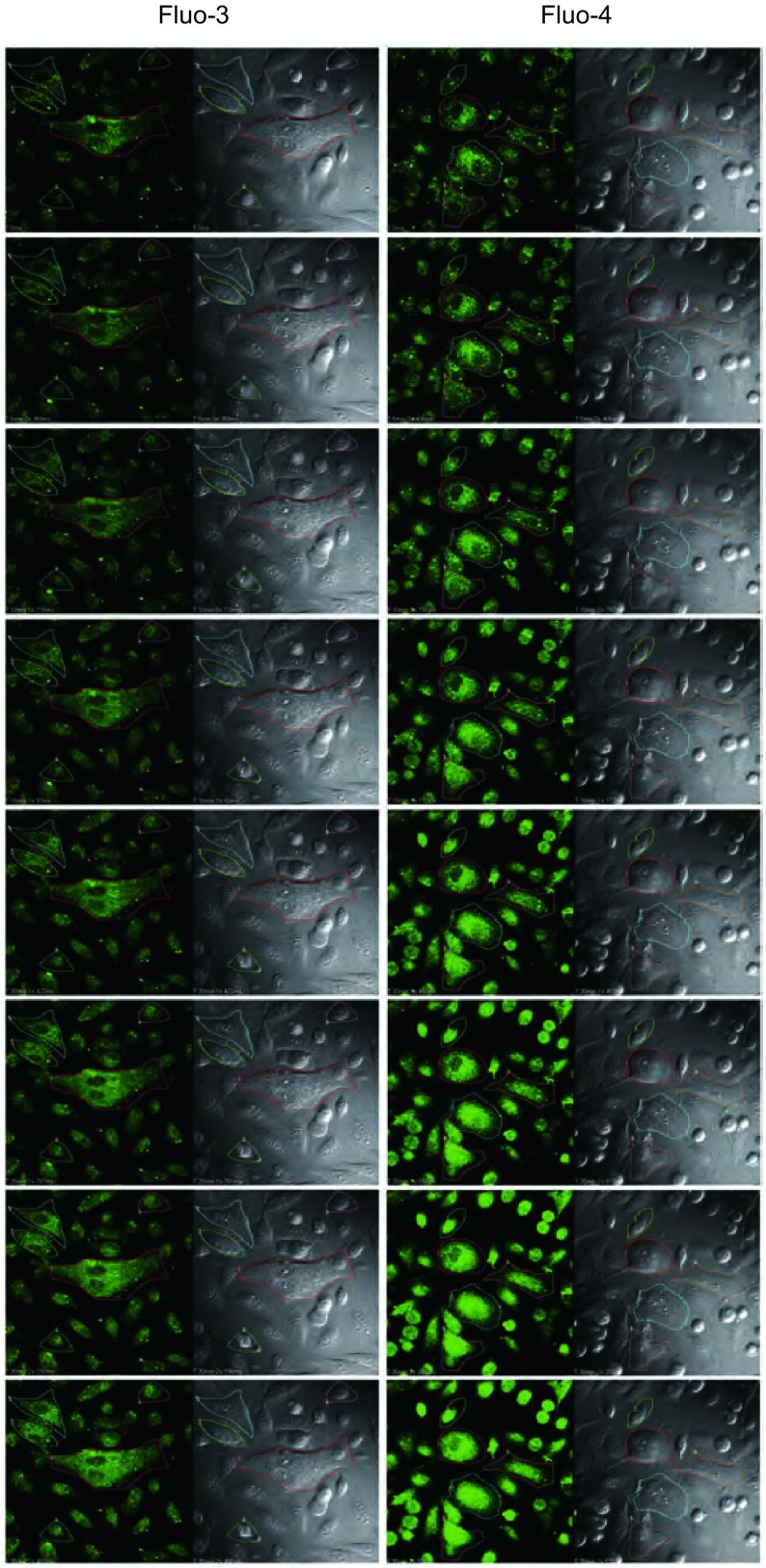
H_2_O_2_刺激后，选定细胞的fluo-3或fluo-4荧光强度变化曲线。145 mm×50 mm（300×300 DPI）。 The fluorescence intensity curve of selected cells loaded with fluo-3 or fluo-4 after H_2_O_2_ stimulation. 145 mm×50 mm (300×300 DPI).

**3:**
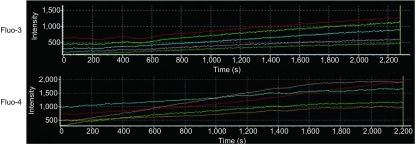
H_2_O_2_刺激后，实时观察细胞中fluo-3和fluo-4荧光强度变化。141 mm×305 mm（300×300 DPI）。 The real-time observation of the fluorescence intensity in selected cells loaded with fluo-3 or fluo-4 after H_2_O_2_ stimulation. 141 mm×305 mm (300×300 DPI).

### 2.3 H_2_O_2_诱导细胞凋亡

细胞经H_2_O_2_刺激后固定，DAPI染色后发现，凋亡细胞呈现核固缩且染色加深，核染色质聚集于核膜一边，或核碎裂成大小不等的圆形小体（图 4B），而对照组细胞核形态规则、染色均匀，呈蓝白色荧光（图 4A）。结果显示与对照组细胞相比，H_2_O_2_处理的细胞凋亡率明显增加（12.2%±2.3% *vs* 33.4%±3.2%），有统计学意义（*P* < 0.001）。

**4:**
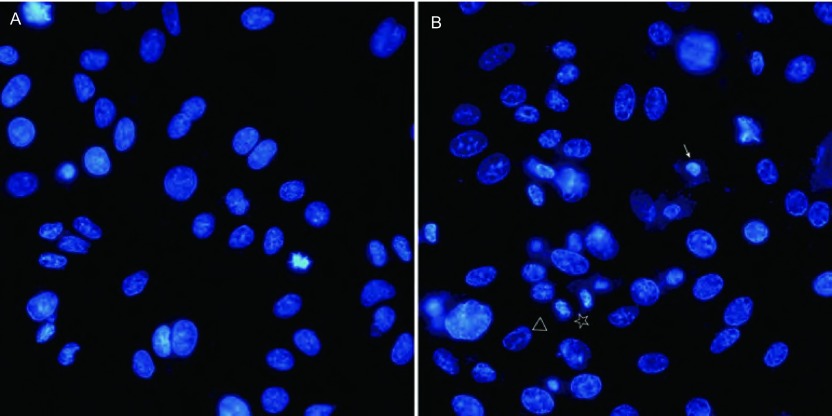
H_2_O_2_促进A549细胞凋亡。A:未经H_2_O_2_处理的细胞；B: H_2_O_2_处理的细胞，可见核固缩（→）、染色质边集（△）以及核碎裂（☆）。65 mm×32 mm（300×300 DPI）。 H_2_O_2_ promoted the apoptosis of A549 cells. H_2_O_2_: cells without H_2_O_2_ treatment; B: karyopyknosis (→), chromatic condensation (△) and nuclear fragmentation (☆) were observed in H_2_O_2_ treated cells. 65 mm×32 mm (300×300 DPI).

## 3 讨论

本研究在对Ca^2+^信号实时观察的过程中发现，较高浓度的H_2_O_2_（50 mM）诱导细胞内[Ca^2+^]_i_迅速升高。由于细胞外液不含有Ca^2+^，因此[Ca^2+^]_i_升高可能是由于细胞的氧化应激反应，导致细胞内钙库（如内质网）释放造成的。有研究^[9]^表明，H_2_O_2_诱导细胞凋亡与钙超载密切相关，氧化应激诱导细胞凋亡的分子机制非常复杂，涉及很多信号转导通路，包括经典的线粒体途径^[10]^和死亡受体途径^[11]^，以及机制仍不太明确的内质网途径^[12]^。近年来，对内质网凋亡途径的研究发现，内质网应激导致的非折叠蛋白反应（unfolded protein response, UPR）扮演着重要角色。研究^[13]^显示，细胞的氧化应激损伤，导致内质网释放大量Ca^2+^，同时伴有内质网应激，引发UPR，用于ER正常功能的重建。如果细胞内Ca^2+^浓度持续升高，内质网应激持续时间较长或非常严重，将激活依赖Ca^2+^的激酶和磷酸酶^[14]^，如calpain、caspase-12和caspase-3级联反应，最终导致细胞凋亡^[15]^。本研究发现，肺癌细胞A549在较高浓度的H_2_O_2_作用下，细胞内Ca^2+^浓度明显升高，同时发现细胞短时间内即发生凋亡，推测可能与内质网应激有关，calpains或caspases信号通路是否被Ca^2+^激活导致细胞凋亡，还有待于进一步深入研究。本研究中，可能由于肿瘤细胞的生理Ca^2+^含量较低，或实时观察时间有限，预实验时使用较低浓度的H_2_O_2_时（5 mM, 10 mM）时，没有观察到明显的[Ca^2+^]_i_变化，因此我们采用较高浓度的H_2_O_2_刺激细胞，低浓度H_2_O_2_对A549细胞其他生物学行为的影响还有待于进一步研究。

**1:** H_2_O_2_刺激前后，选定细胞中fluo-3和fluo-4的平均荧光强度值 The mean value of fluorescence intensity in selected cells loaded with fluo-3 or fluo-4 before and after H_2_O_2_ treatment

	Fluo-3			Fluo-4	
	Mean fluorescence intensity	Standard deviation		Mean fluorescence intensity	Standard deviation
Before stimulation	290.42	151.34		594.71	261.41
After stimulation	867.32	415.66		1558.48	437.54

Fluo-3和fluo-4是目前实验室中常用的Ca^2+^荧光染料，与Ca^2+^特异性结合后荧光强度明显增加。本研究对fluo-3和fluo-4在H_2_O_2_诱导的A549细胞凋亡过程中监测[Ca^2+^]_i_变化的应用情况进行了比较。结果发现，在相同的负载浓度、孵育时间、细胞密度、刺激因素和图像采集条件下，fluo-4的荧光强度更强，大约是fluo-3的2倍左右，这提示我们当细胞内Ca^2+^信号较强时，用fluo-3或fluo-4都可以观察到明显的荧光强度的变化，但如果细胞内Ca^2+^信号较弱时，使用fluo-4探针可能更具优势。细胞经H_2_O_2_处理后，fluo-3荧光强度的变化范围大于fluo-4，可能由于荧光探针的Kd值不同，具有较大Kd值的fluo-3与Ca^2+^的亲和力较低，适合于检测较宽范围的[Ca^2+^]_i_变化。我们还发现，采用荧光探针fluo-3或fluo-4，通过公式计算测定的细胞内[Ca^2+^]_i_变化范围不是很一致，可能与选择的细胞有关，不同的细胞对H_2_O_2_刺激的反应不同，再多重复几次实验，可能会得到比较一致的结果。

Fluo-3和fluo-4进入细胞的方式均为酯负载法。Fluo-3和fluo-4与具有细胞膜通透性的乙酰甲酯（AM）相连形成Fluo-3 AM和fluo-4 AM复合物，穿过细胞膜后在细胞内被非特异性酯酶水解生成相应的Ca^2+^荧光探针，与Ca^2+^结合检测细胞内[Ca^2+^]_i_变化。我们在实验过程中发现，含血清的培养基会影响fluo-3和fluo-4的负载，在激光共聚焦显微镜下观察荧光非常微弱，采集不到良好的荧光信号，这可能是由于含血清的培养基能阻止荧光染料进入细胞。使用标准的无钙细胞外液，由于没有血清和培养液的营养支持，对细胞状态和活性的影响较大，细胞可能在观察过程中逐渐皱缩、脱壁或凋亡，不能对细胞进行长时间检测。

近几年还出现了荧光强度更强、检测范围更宽、更灵敏的Ca^2+^荧光染料，如fluo-5F、fluo-5N、fluo-4FF，均为fluo-4的类似物，但与Ca^2+^结合的亲和力较低，更适合于检测1 μM-1 mM范围内的Ca^2+^水平变化。Fluo-3和fluo-4测定的Ca^2+^饱和浓度为≥5 μM，当[Ca^2+^]_i_升高超过5 μM时，即使有更多的Ca^2+^出现在细胞中，fluo-3和fluo-4也检测不到，而fluo-5F、fluo-5N、fluo-4FF测定的Ca^2+^饱和浓度为≥1 mM，这些新的Ca^2+^探针可以检测到更多的Ca^2+^，在检测高水平的Ca^2+^信号时更具优势。Fluo-5F、fluo-4FF的Kd值分别为2.3 μM和9.7 μM，而fluo-5N的Kd值高达90 μM，适合检测更高浓度的Ca^2+^变化^[16]^。研究表明应根据细胞内[Ca^2+^]_i_的变化水平选择合适的Ca^2+^荧光探针，当待测Ca^2+^浓度在荧光染料Kd值的0.1倍-10倍范围之内时其荧光强度与[Ca^2+^]_i_呈良好的线性关系，检测结果最准确。

Fluo-3和fluo-4及其类似物均为化学合成的Ca^2+^荧光染料，与Ca^2+^有较高的亲和力，通过负载方式很容易进入细胞，但不能在亚细胞水平精确定位，而且可能出现光漂白现象，长时间观察时对细胞活性有影响。除了Ca^2+^荧光染料，近年来还出现了水母发光蛋白和Cameleon等^[17]^基于生物发光的Ca^2+^荧光蛋白探针，需要通过基因转染方式进入细胞，与细胞器特异性基因连接表达重组蛋白，可实现精确的亚细胞定位，长时间观察其荧光强度稳定不会淬灭。目前我们已应用Ca^2+^荧光蛋白Cameleon YC3.6对H_2_O_2_刺激后A549细胞中[Ca^2+^]_i_进行了测定^[18]^，但在比较化学合成的Ca^2+^荧光探针或Ca^2+^荧光蛋白这两种不同的检测方法方面，还有待于进一步研究。我们应根据实验目的、细胞种类、刺激因素以及检测条件选择合适的Ca^2+^荧光探针，以帮助我们深入研究不同刺激条件下肿瘤细胞内[Ca^2+^]_i_变化，Ca^2+^信号转导的相关分子机制，以及对肿瘤细胞生物学行为的影响。
